# Enhanced magnetocaloric effect in Ni-Mn-Sn-Co alloys with two successive magnetostructural transformations

**DOI:** 10.1038/s41598-018-26564-5

**Published:** 2018-05-29

**Authors:** Xuexi Zhang, Hehe Zhang, Mingfang Qian, Lin Geng

**Affiliations:** School of Materials Science and Engineering, Harbin Institute of Technology, Harbin, 150001 China

## Abstract

High magnetocaloric refrigeration performance requires large magnetic entropy change *ΔS*_*M*_ and broad working temperature span *ΔT*_*FWHM*_. A fourth element doping of Co in ternary Ni-Mn-Sn alloy may significantly enhance the saturation magnetization of the alloy and thus enhance the *ΔS*_*M*_. Here, the effects of Co-doping on the martensite transformation, magnetic properties and magnetocaloric effects (MCE) of quaternary Ni_47−x_Mn_43_Sn_10_Co_x_ (x = 0, 6, 11) alloys were investigated. The martensite transformation temperatures decrease while austenite Curie point increases with Co content increasing to x = 6 and 11, thus broadening the temperature window for a high magnetization austenite (13.5, 91.7 and 109.1 A·m^2^/kg for x = 0, 6 and 11, respectively). Two successive magnetostructural transformations (A → 10 M and A → 10 M + 6 M) occur in the alloy x = 6, which are responsible for the giant magnetic entropy change *ΔS*_*M = *_29.5 J/kg·K, wide working temperature span *ΔT*_*FWHM*_ = 14 K and large effective refrigeration capacity *RC*_*eff*_ = 232 J/kg under a magnetic field of 5.0 T. These results suggest that Ni_40.6_Mn_43.3_Sn_10.0_Co_6.1_ alloy may act as a potential solid-state magnetic refrigerant working at room temperature.

## Introduction

Over the last decade, Ni-Mn-X (X = Sn, In and Sb) metamagnetic shape memory alloys (MSMAs) have attracted significant interests due to their potential applications as magnetic refrigeration materials near room temperature. These materials, based on magnetocaloric effect (MCE), are considered to be environmentally friendly and cost-effective refrigerants because of their large refrigeration capacity (*RC*) which is comparable to those of expensive rare-earth-containing MCE compounds^1^. In Ni-Mn-X-based (X = Sn, In and Sb) alloys, the metamagnetic structural transition from the weak-magnetic martensite to ferromagnetic austenite under an applied magnetic field leads to an inverse MCE^2–4^, whereas the magnetic transition of the austenite phase is responsible for a conventional MCE^5–7^. Clearly, the metamagnetic transition is driven by the Zeeman energy *E*_*zeeman*_ = *μ*_0_*HΔM*, where *ΔM* and *μ*_0_*H* represent the magnetization difference between martensite/austenite phases and the applied magnetic field, respectively. So, enhanced *ΔM* is in favor of large *E*_*zeeman*_, which is responsible for the high magnetic entropy change (*ΔS*_*M*_) and wide working temperature span (*ΔT*_*FWHM*,_ defined as the full width at half maximum of the magnetic entropy peak)^8^, where the *ΔT*_*FWHM*_ is crucial for magnetic refrigeration applications in the case of inverse MCE^9,10^.

In Ni-Mn-X off-stoichiometric MSMAs, the *ΔM* can be enhanced by composition tuning^11,12^, heat treatment^13,14^ and doping^15,16^. It has been reported that high-Mn content Ni-Mn-X alloys exhibited enhanced *ΔM* because the magnetization of the austenite was mainly attributed to the ferromagnetic interaction between the neighboring Mn-Mn atoms^17^. For instance, the austenite of Mn-rich Ni_43_Mn_46_Sn_11_ alloy^18^ shows a higher magnetization of 68 A·m^2^/kg under a magnetic field of 5.0 T, compared to that of 39 A·m^2^/kg for Ni_46_Mn_43_Sn_11_ alloy^19^. Heat treatment is also a useful method to modulate the grain constraint in order to control the microstructure, martensite transformation (MT) temperature and magnetic properties of the ferromagnetic MSMAs (FMSMAs)^20,21^. Doping of non-ferromagnetic elements^22–24^ such as Cu, Al and Ti can enhance *ΔM* effectively by tuning the valence electron concentration *e*/*a* or changing the unit cell volume, which mainly changes the MT temperatures. Of note is that the doping of ferromagnetic elements such as Fe and Co can effectively improve the *ΔM*. For instance, the substitution of Fe for Ni can simultaneously enhance the magnetic-field-induced reverse martensitic transformation and mechanical properties of Ni-Mn-Sn^25–27^. Previous studies suggested that Co substitution for Ni in Ni-Mn-X alloys can effectively improve the austenite saturation magnetization and decrease the martensite magnetization^28–30^, which was mainly due to the following four aspects: 1) The introduction of Co acted as a “ferromagnetic activator” to induce the magnetic moments of the nearest neighboring Mn-Mn atoms aligning in a ferromagnetic order^31,32^, 2) Upon substitution of Co for Ni in Ni-Mn-X alloys, the austenite Curie point (*T*_*C*_^*A*^) increased significantly with increasing Co concentration, which broadened the temperature window of the high magnetization austenite^33^, 3) The doping of Co can increase the Curie point and saturation magnetization of the Ni-Mn-X alloys, which accordingly increases the *E*_*zeeman*_^34^, 4) Co atoms at Ni site contribute a much larger magnetic moment (~1.0 µ_B_) compared to that of Ni (~0.3 µ_B_) in the austenite^35^.

The enlargement of *ΔT*_*FWHM*_ may be realized by two successive magnetostructural transformations, which have been found in some ferromagnetic alloys. For example, rare earth containing low temperature magnetic refrigeration compounds, such as TbMn_2_Si_2_^36^, ErGa^37^, DyB_2_^38^, HoPdIn^39^, may exhibit a two successive magnetic transitions behavior deriving from the coupling of spin-reorientation temperature (TSR) and Curie temperature (*T*_*c*_); Two successive *ΔS*_*M*_ peaks with the same sign, associated with a first-order martensite transformation (MT) and an intermediate martensite transformation (IMT), have also been discovered in some Ni-Mn-X alloys after composition tuning or under external pressure^40–42^. These two adjacent transformations lead to a partially overlap of the refrigerant temperature intervals, yielding an improved refrigerating capacity. Here, we reported the MT, magnetic transition and MCE of Ni_47−x_Mn_43_Sn_10_Co_x_ (x = 0, 6, 11) alloys. Two successive magnetostructural transformations from austenite phase to two different modulated martensite phases, i.e. A → 10 M and A → 10 M + 6 M, were demonstrated in the Ni_40.6_Mn_43.3_Sn_10.0_Co_6.1_ (Co6) alloy induced by an external magnetic field. As a consequence, a large magnetic entropy change *ΔS*_*M*_ of 29.5 J/kg·K with a wide *ΔT*_*FWHM*_ of ~14 K attributed to the occurrence of successive magnetostructural transformations and a strong metamagnetic transition behavior were revealed in the Co6 alloy.

## Results and Discussion

### Microstructure of the Ni_40.6_Mn_43.3_Sn_10.0_Co_6.1_ (Co6) alloy

**Figure 1:**
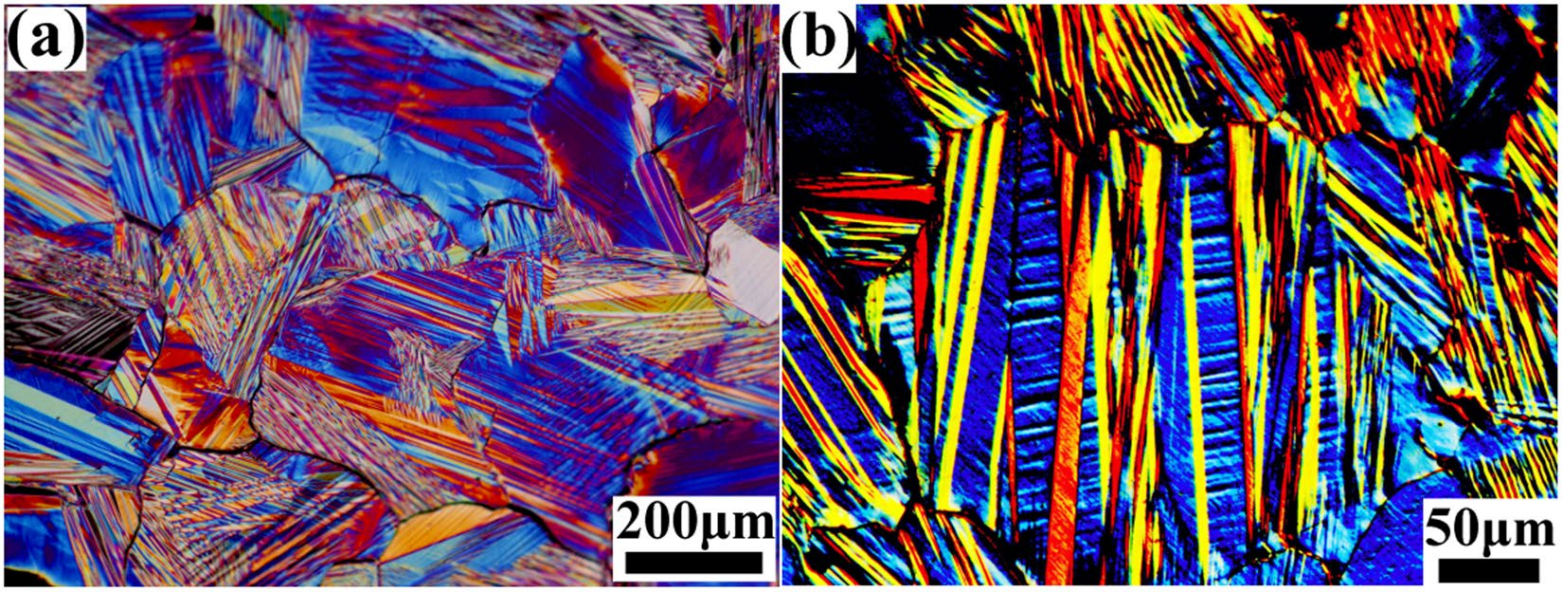
Optical microscope (OM) images showing the grains and martensite twin morphology in the Co6 alloy. (**a**) and (**b**) are low and high magnification images, respectively.

The typical microstructure of the martensite phase of the Co6 alloy is shown in Fig. 1, where the sample was firstly kept in the ice water for several minutes to reach the martensite state. The microstructure is very similar to those modulated martensite structures reported in some ferromagnetic shape memory alloys^43^. Many cracks distributed along the grain boundaries were observed, as shown in Fig. 1a, implying the brittleness of the polycrystalline alloy. In addition, the martensite morphology is plate-like, which can be recognized by the straight twin boundaries of each plate. Fine twins with thickness of 2–10 μm exist inside the broad martensite plates, as shown in Fig. 1b. Previous report^44^ showed that different martensite structures possess different morphologies for Ni-Mn-Sn alloys, where the 10 M martensite exhibited broad plate morphology, 14 M martensite a fine form and the unmodulated structure (L1_0_) in between. The co-existence of the martensite twins with different widths in the present work implied the existence of different martensite structures in Co6 alloy at room temperature (RT). Detailed martensite structure of the present alloy will be analyzed by XRD and discussed in the later sections.

### Martensite transformation of Ni_47−x_Mn_43_Sn_10_Co_x_ (x = 0, 6, 11) alloys

**Figure 2:**
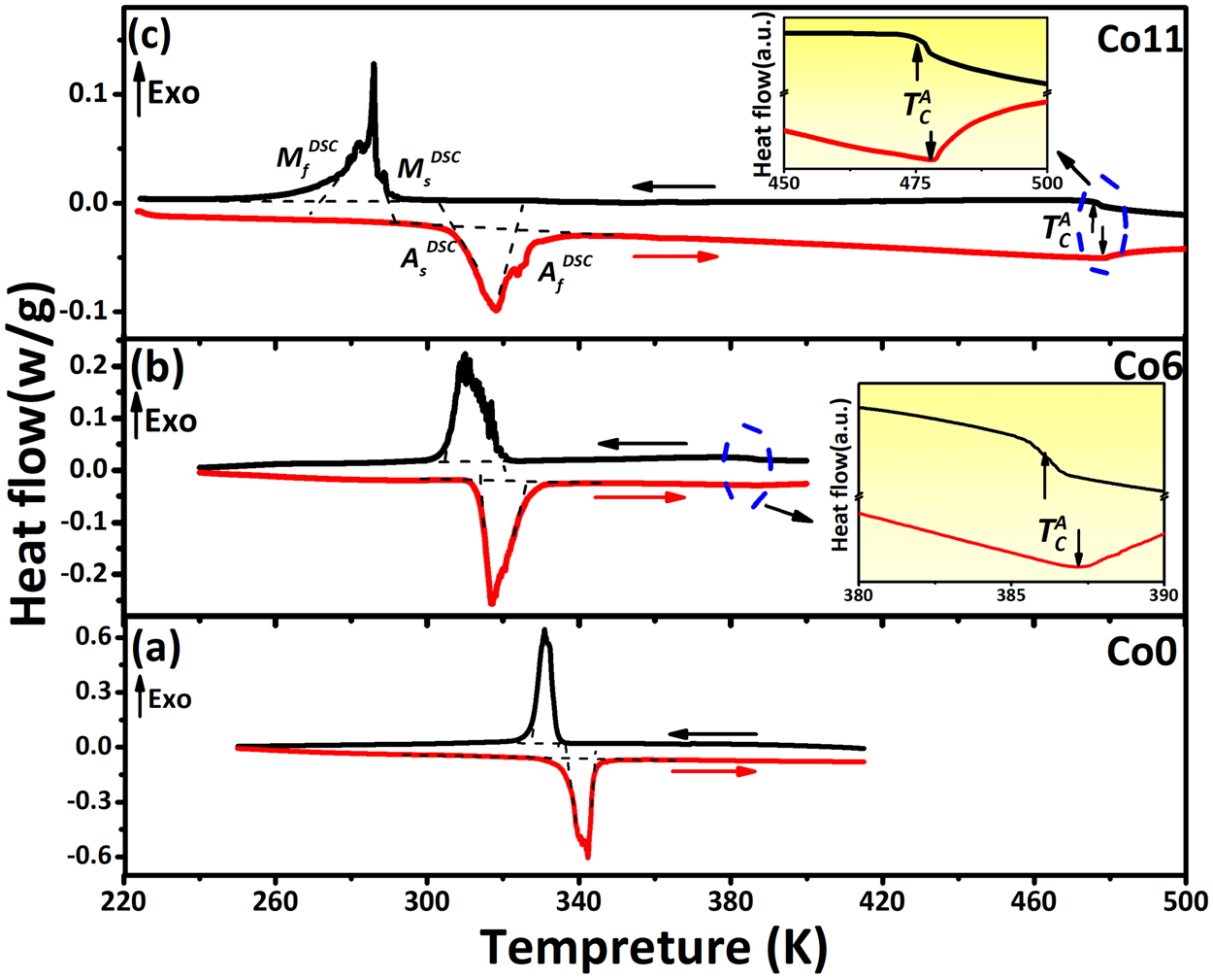
Differential scanning calorimetry (DSC) plots of the annealed Ni_47−x_Mn_43_Sn_10_Co_x_ (x = 0, 6, 11) alloys at heating and cooling rates of 10 K/min. (**a**) Co0, (**b**) Co6 and (**c**) Co11. Insets in (**b**) and (**c**) are enlarged images showing the magnetic transitions around the Curie point.

Figure 2 shows the DSC curves of Ni_47−x_Mn_43_Sn_10_Co_x_ (x = 0, 6, 11) alloys in the temperature range 220–500 K. The strong exothermic and endothermic peaks produced by the forward and reverse MT correspond to the starting and finishing temperatures *M*_*s*_^*DSC*^, *M*_*f*_^*DSC*^, *A*_*s*_^*DSC*^ and *A*_*f*_^*DSC*^, respectively, determined by the intersection of the base-line and tangent line. These temperatures are summarized in Table 1 together with the values of the valence electrons per atom (*e*/*a*). With the addition of Co element, a monotonic decrease in the MT temperatures occurs, as shown in Table 1. Generally, the change in MT temperatures can be interpreted from the following two aspects. Firstly, the *e*/*a* dependence of MT temperatures has been found to increase monotonically in Ni-Mn-based Heusler alloys^45,46^. It has been supposed that the L2_1_ austenite is stabilized because its Fermi surface just touches the Brillouin zone boundary^47^. With *e*/*a* increasing and thus the Fermi surface overlapping the Brillouin zone, the L2_1_ austenite structure becomes instable, which induces the occurrence of the MT^47^. Here, the values of *e*/*a* are the concentration-weighted sum of *s*, *d*, and *p* valence electrons, i.e. 10 (3*d*^8^4*s*2) for Ni, 7 (3*d*^5^4*s*2) for Mn, 4 (5*s*^2^5*p*2) for Sn and 9 (3*d*^7^4*s*^2^) for Co, respectively. The calculated *e*/*a* decreases with increasing Co substitution of Ni mainly because the valence electrons of Co is less than Ni, thus decreasing the MT temperatures of the alloy, which is in good agreement with the *e*/*a* dependence rule^45,46^. On the other hand, the atom size effect, originating from the unit cell expansion of the austenite, is not favorable to the occurrence of MT due to changes of the relative positions between the Fermi surface and Brillouin zone^29^. The substitution of larger Co atoms (atomic radius r = 0.126 nm) for smaller Ni atoms (r = 0.125 nm) causes a slight expansion of the austenitic unit cell. Both factors stabilize the austenite phase and therefore decrease the MT temperatures.

**Table 1:** Martensite transformation temperatures (*A*_*s*_^*DSC*^, *A*_*f*_^*DSC*^, *M*_*s*_^*DSC*^, *M*_*f*_^*DSC*^), Curie point (*T*_*C*_^*A*^), transformation enthalpy change (*ΔH*_*endo*_ and *ΔH*_*exo*_) and transformation entropy change (*ΔS*_*tr*_^*endo*^ and *ΔS*_*tr*_^*exo*^) during heating/cooling process obtained by DSC curves of Ni_47−x_Mn_43_Sn_10_Co_x_ (x = 0, 6, 11) alloys.

Alloys	Transformation temperatures, K					*ΔH*_*endo*_, J/g	*ΔS*_*tr*_^*endo*^, J/kg·K	*ΔH*_*exo*_, J/g	*ΔS*_*tr*_^*exo*^, J/kg·K
	*A*_*s*_^*DSC*^	*A*_*f*_^*DSC*^	*M*_*s*_^*DSC*^	*M*_*f*_^*DSC*^	*T*_*C*_^*A*^				
Co0^1*^	336	344	334	327	—	16.5 ± 0.2	48.5 ± 0.5	16.9 ± 0.1	51.1 ± 0.3
Co6^2*^	314	326	310	304	387	10.8 ± 0.06	33.7 ± 0.2	9.9 ± 0.06	32.2 ± 0.2
Co11^3*^	307	323	289	271	476	5.5 ± 0.2	17.7 ± 0.6	4.7 ± 0.2	16.9 ± 0.6

^1*^Co0 - Ni_45.6±0.2_Mn_43.6±0.1_Sn_10.8±0.1_, ^2*^Co6 - Ni_40.6±0.1_Mn_43.3±0.1_Sn_10.0±0.04_Co_6.1±0.04_, ^3*^Co11 - Ni_35.7±0.2_Mn_44.1±0.2_Sn_9.2±0.06_Co_11.0±0.1_ (data indicate atomic percent, at. %).

**Figure 3:**
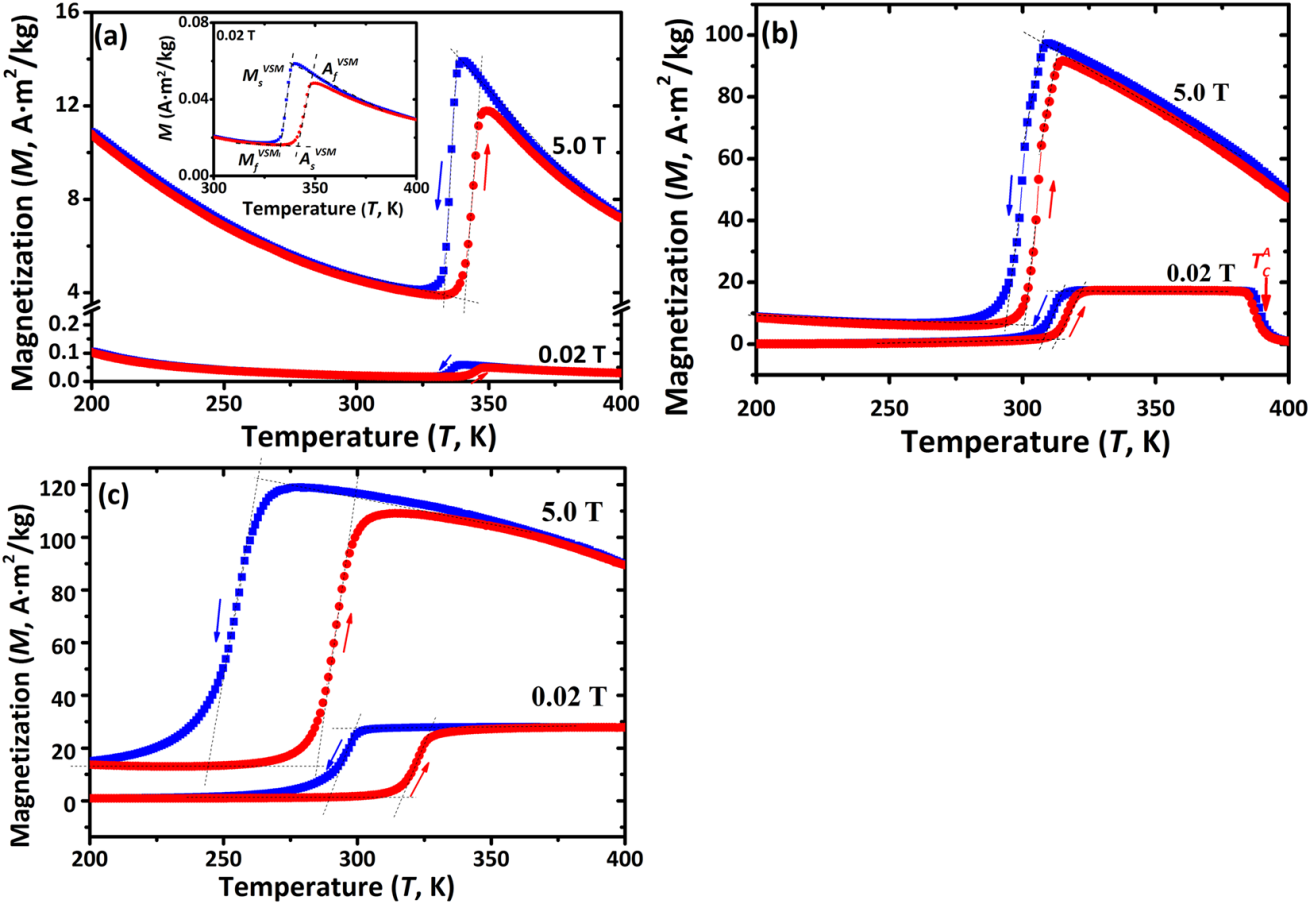
Temperature dependence of the magnetization (*M-T*) curves under external magnetic fields of 0.02 and 5.0 T for the alloys of (**a**) Co0, (**b**) Co6 and (**c**) Co11. The inset in (**a**) shows an enlarged *M-T* curves of Co0 alloy under a magnetic field of 0.02 T.

A further feature of weak shoulder thermal effect (*T*_*C*_^*A*^) is also observed for sample Co6 and Co11, as shown in the inset in Fig. 2b,c, respectively, which corresponds to the magnetic transition from ferromagnetism to paramagnetism of the austenite during cooling process. But the *T*_*C*_^*A*^ point was not found for Co0 probably because the magnetic transition occurs below MT temperatures leading to the extremely low magnetization of the austenite (as shown in Fig. 3a). Therefore, it can be deduced that the substitution of Co for Ni in Ni-Mn-Sn alloys effectively increases the *T*_*C*_^*A*^, which is consistent with refs^29,30^. The comparison of the transformation entropy changes during heating (*ΔS*_*tr*_^*endo*^) and cooling (*ΔS*_*tr*_^*exo*^) process are also listed in Table 1 (*ΔS*_*tr*_^*endo*^ = *ΔH*_*endo*_/*T*_*t*_, where *T*_*t*_ = (*A*_*s*_^*DSC*^ + *A*_*f*_^*DSC*^)/2; *ΔS*_*tr*_^*exo*^ = *ΔH*_*exo*_/*T*_*t*_, where *T*_*t*_ = (*M*_*s*_^*DSC*^ + *M*_*f*_^*DSC*^)/2). *ΔH*_*endo*_ and *ΔH*_*exo*_, denote as the enthalpy changes during the inverse and direct MT, was obtained from the area between the DSC peaks and the baseline. The results show that the difference between *ΔS*_*tr*_^*endo*^ and *ΔS*_*tr*_^*exo*^ are very small (≤2.6 J/kg·K).

Figure 3 shows the field-heating (FH) and field-cooling (FC) magnetization *vs* temperature plots (*M-T*) of Ni_47−x_Mn_43_Sn_10_Co_x_ (x = 0, 6, 11) alloys measured under magnetic fields of 0.02 and 5.0 T. The magnetization change (*ΔM*) in Co0 between austenite and martensite phases during MT is very small (Fig. 3a) because the phase transformation temperatures are higher than the magnetic transition point (*T*_*tr*_ > *T*_*C*_^*A*^) and thus MT occurs in a weak-magnetic state. Furthermore, the metamagnetic transition behavior is also weak in Co0 alloy, i.e. its MT temperatures shift little to lower temperatures with increasing magnetic field (*ΔA*_*s*_*/ΔH* = −0.2 K/T, as shown in Table 2 based on Fig. 3a), which is unfavorable to MCE^2^. In contrast, both Co6 and Co11 alloys undergo the transformation between a weak-magnetic martensite and a ferromagnetic austenite (Fig. 3b,c), leading to higher *ΔM*.

**Table 2:** Martensite transformation temperatures and magnetic properties obtained by vibrating sample magnetometer (VSM) for Ni_47−x_Mn_43_Sn_10_Co_x_ (x = 0, 6, 11) alloys under magnetic fields of 0.02 and 5.0 T. *M*_*A*_ and *M*_*M*_ are magnetizations of the austenite and martensite, respectively.

Alloys	Magnetic field, T	Transformation temperatures, K					Magnetization^2^*, A·m^2^/kg			*ΔA*_*s*_*/ΔH*, K/T
		*A*_*s*_^*VSM*^	*A*_*f*_^*VSM*^	*M*_*s*_^*VSM*^	*M*_*f*_^*VSM*^	*ΔT*_*hys*_^1^*	*M*_*M*_	*M*_*A*_	*ΔM*^3^*	
Co0	0.02	341	349	338	333	9.5	0.02	0.06	0.04	−0.2
	5.0	340	348	338	332	9	4.3	13.5	9.2	
Co6	0.02	313	323	315	308	6.0	0.1	17.4	17.3	−2.6
	5.0	300	316	306	294	6.0	6.0	91.7	85.7	
Co11	0.02	316	328	301	289	27	0.9	27.9	27.0	−6.6
	5.0	283	301	263	244	38.5	13.0	109.1	96.1	

^1^*ΔT_*hys*_* = *[(*A*_*f*_ + *A*_*s*_) − (*M*_*s*_ + *M*_*f*_)]/2. ^2*^Data obtained during heating process. ^3*^ΔM = M_*A*_ − *M*_*M*_.

Table 2 collected the MT and magnetic transition temperatures of the Ni_47−x_Mn_43_Sn_10_Co_x_ (x = 0, 6, 11) alloys. It can be seen that the MT temperatures determined from vibrating sample magnetometer (VSM) under 0.02 T are consistent with those from DSC (Table 1). The thermal hysteresis values *ΔT*_*Hys*_, defined as [(*A*_*f*_ + *A*_*s*_) − (*M*_*s*_ + *M*_*f*_)]/2, are 6.0 K under both 0.02 and 5.0 T in the Co6 alloy, which are much smaller than those of 27 K (0.02 T) and 38.5 K (5.0 T) in the Co11 alloy. As the phase boundary friction strength during MT is responsible for *ΔT*_*Hys*_^48^, this implies that the friction resistance during MT in Co11 alloy is larger than that in Co6 alloy.

It can also be seen from Table 2 that, during heating, the magnetization of the martensite *M*_*M*_ changes little but that of the austenite *M*_*A*_ increases significantly with increasing Co content. As a result, *ΔM* in Co6 and Co11 alloys reaches 85.7 and 96.1 A·m^2^/kg under a magnetic field 5.0 T, respectively, which is much higher than *ΔM* = 9.2 A·m^2^/kg in Co0. The enhancement of *ΔM* favors the magnetic entropy change *ΔS*_*M*_ according to the Maxwell equation and broadens the *ΔT*_*FWHM*_ due to the increase in Zeeman energy^45^. Furthermore, the percentage of MT caused by field-induced metamagnetic transition mainly relies on d*T*_*M*_/d*H*^40^ (*T*_*M*_ denotes the MT equilibrium temperature, as shown in Fig. 3, which can be assessed by the Clausius-Clapeyron magnetic equation *−*d*T*_*M*_*/dH* = *ΔM/ΔS*_*tr*_. Utilizing the values of *ΔM* under 5.0 T (Table 2) and *ΔS*_*tr*_ determined by DSC (Table 1), the calculated *ΔM/ΔS*_*tr*_ is −2.5 and −5.4 K/T for Co6 and Co11, respectively. By contrast, the magnetic-field-induced shift of the reverse MT temperature *ΔA*_*s*_*/ΔH* is −2.6 and −6.6 K/T for Co6 and Co11 alloys, respectively, as listed in Table 2. So, the applied 5.0 T magnetic field may induce a full metamagnetic transition from weak-magnetic martensite to ferromagnetic austenite in Co6 alloy, but only a partial transition in Co11 alloy, meaning that a 5.0 T magnetic field cannot compensate for the thermal energy loss during MT in Co11 alloy^48^. Supposing a negligible difference of heat capacity between martensite/austenite phases, a maximum magnetic entropy change *ΔS*_*M*_^*max*^ ≈ *ΔS*_*tr*_ should be expected under a high *ΔAs/ΔH*^49^. As a result, a sizable inverse MCE in Co6 alloy is expected when taking the *ΔM*, *ΔS*_*tr*_, *ΔA*_*s*_*/ΔH* and *ΔT*_*Hys*_ into account.

**Figure 4:**
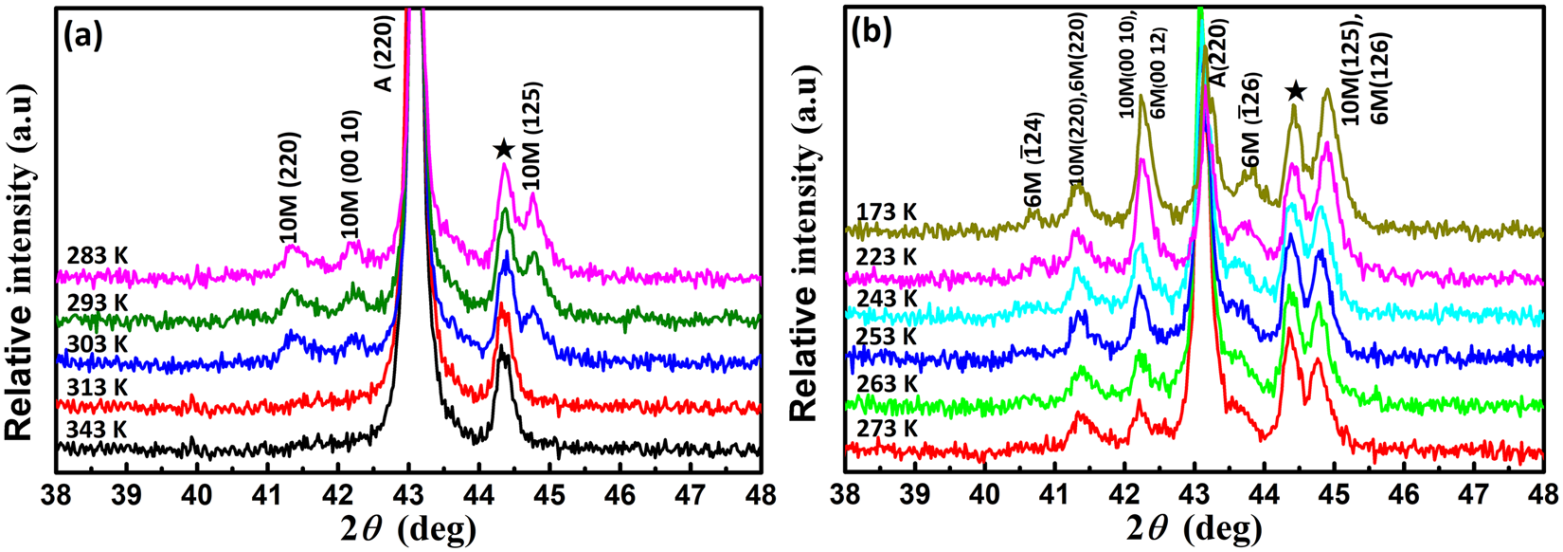
*In-situ* X-ray diffraction (XRD) patterns for Co6 alloy in the range 35° < 2θ < 50° during cooling (**a**) from 343 K to 283 K and (**b**) from 273 K to 173 K, respectively. The diffraction peaks marked by five-pointed star corresponds to the sample stage.

In order to investigate the martensite transformation behavior of the Co6 alloy, X-Ray diffraction (XRD) measurements were performed in the temperature range 343–173 K during cooling process. The data were recorded at a temperature interval 10 K (except for 223 K and 173 K), as shown in Fig. 4. The diffraction peaks marked with five-pointed star are associated with the sample platform (Fig. 4). The martensite peaks were indexed as 10 M and 6 M martensite phases according to the theoretical calculation results^50^ and is displayed in the Supplementary Materials Fig. S2. The alloy exhibits a L2_1_-type austenite structure at high temperatures. With decreasing temperature from 343 to 283 K, the peaks corresponding to the 10 M martensite occur starting at 303 K, as shown in Fig. 4a. As the temperature further decreases, several peaks associated with the 6 M martensite appear at 273 K (much lower than *M*_*f*_* = *304 K from Table 1), as shown in Fig. 4b. It can also be seen that the peak intensity of 6 M martensite becomes stronger with decreasing temperature. On the other hand, the peak intensity of 10 M martensite still keeps increasing with decreasing temperature after the occurrence of the 6 M martensite, implying the co-existence of the A, as shown 6 M transitions and the mixed A + 10 M + 6 M phase state during the MT process of the Co6 alloy. Such mixed state of 10 M and 6 M martensite phases has also been found in Ni_43_Co_7_Mn_39_Sn_11_^51^ and Ni_37_Co_11_Mn_43_Sn_9_^50^ alloys. It is worth noting that the peak (220)_A_ of the L2_1_-type austenite can still be observed at temperature as low as 173 K (*see* Fig. 4b), indicating that an incomplete MT with residual austenite even at 173 K. This phenomenon is consistent with some other Ni-Mn-based alloys^52,53^, implying that the complete MT might be difficult to be achieved supposing that slightly inhomogeneous composition distribution exists in the bulk alloy. Furthermore, the two successive magnetostructural transition processes (A → 10 M and A → 10 M + 6 M) in the present alloy are essentially different from the two successive martensite (MT) and intermartensite transition (IMT) occur in some other types of FMSMAs such as Ni_55.8_Mn_18.1_Ga_26.1_^40^, Ni_50_Mn_34_In_12_Sb_4_^54^ and Ni_49−x_Cu_x_Mn_38_Sn_13_ (0.5 < x < 2)^55^ alloys.

In addition, the XRD pattern measured at 343 K (*A*_*f*_ = 326 K) is shown in Supplementary Materials Fig. S1b. Single austenite phase is detected without any secondary phases, which is consistent with the back scattered electron (BSE) micrograph observations (Supplementary Materials Fig. S1a). The result is consistent with that in Ni_34_Co_8_Mn_50_Sn_8_^56^ and Ni_37_Co_11_Mn_43_Sn_9_^50^ after annealing. Secondary phases were observed in Ni_42_Co_8_Mn_39_Sn_11_^57^ and Ni_43_Co_7_Mn_39_Sn_11_^58^ after annealing at 1173 K, however, the excessive Mn content in the present annealed Ni-Mn-Sn-Co alloys may have delayed the occurrence of secondary phase.

### Magnetic properties of the Ni_40.6_Mn_43.3_Sn_10.0_Co_6.1_ (Co6) alloy

**Figure 5:**
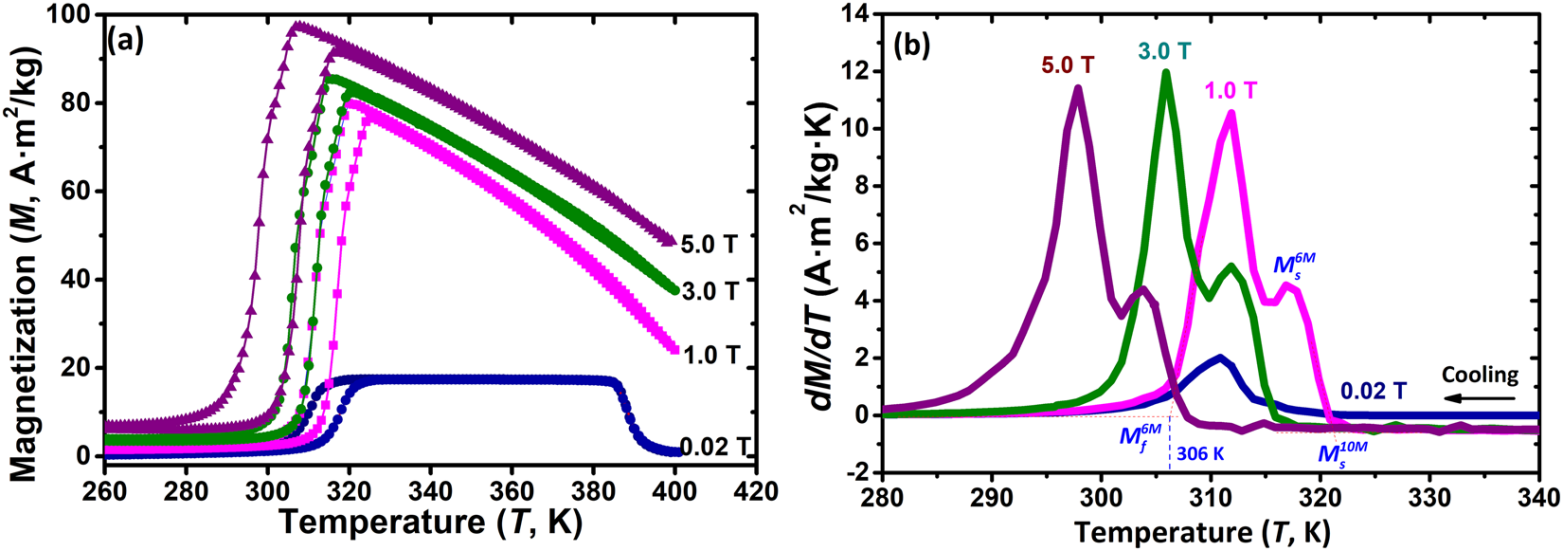
Magnetization curves as a function of temperature under magnetic fields of 0.02, 1.0, 3.0 and 5.0 T in the Co6 alloy. (**a**) Isofield magnetization (*M*-*T*) curves, (**b**) First-derivative plots of *M-T*.

The temperature dependent FH/FC magnetization curves and the *dM/dT* vs *T* of the Co6 alloy recorded at magnetic fields 0.02, 1.0, 3.0 and 5.0 T are shown in Fig. 5a,b. It can be observed from Fig. 5a that slope changes in the FH/FC curves exist around MT temperatures at magnetic fields above 1.0 T. To clarify this phase transformation character, the first-derivative of the FC magnetization curves with respect to temperature (d*M*/*dT* - *T*) was plotted in Fig. 5b. Upon cooling, two successive maxima occur under magnetic fields of 1.0, 3.0 and 5.0 T, while only one inflection peak emerges at 0.02 T. Additionally, the d*M*/d*T* - *T* curves show that *M*_*s*_^*10M*^ and *M*_*s*_^*6M*^, denote as the 10 M and 6 M transformation starting temperatures, respectively, decrease with increasing magnetic field (i.e. the 10M and 6M change from 321 and 315 K under *μ*_*0*_*H* = 1.0 T to 308 and 301 K under *μ*_*0*_*H* = 5.0 T, respectively). This phenomenon probably indicate that the two successive magnetostructural transformations (A → 10 M + 6 M and A → 10 M) can be induced by an external magnetic field, which is still an open question and will be studied in the near future. Compared to the XRD patterns during cooling from 343 to 173 K (*see* Fig. 4), the magnetic-field-induced inverse martensite transformation is much easier than the temperature-field-induced one.

**Figure 6:**
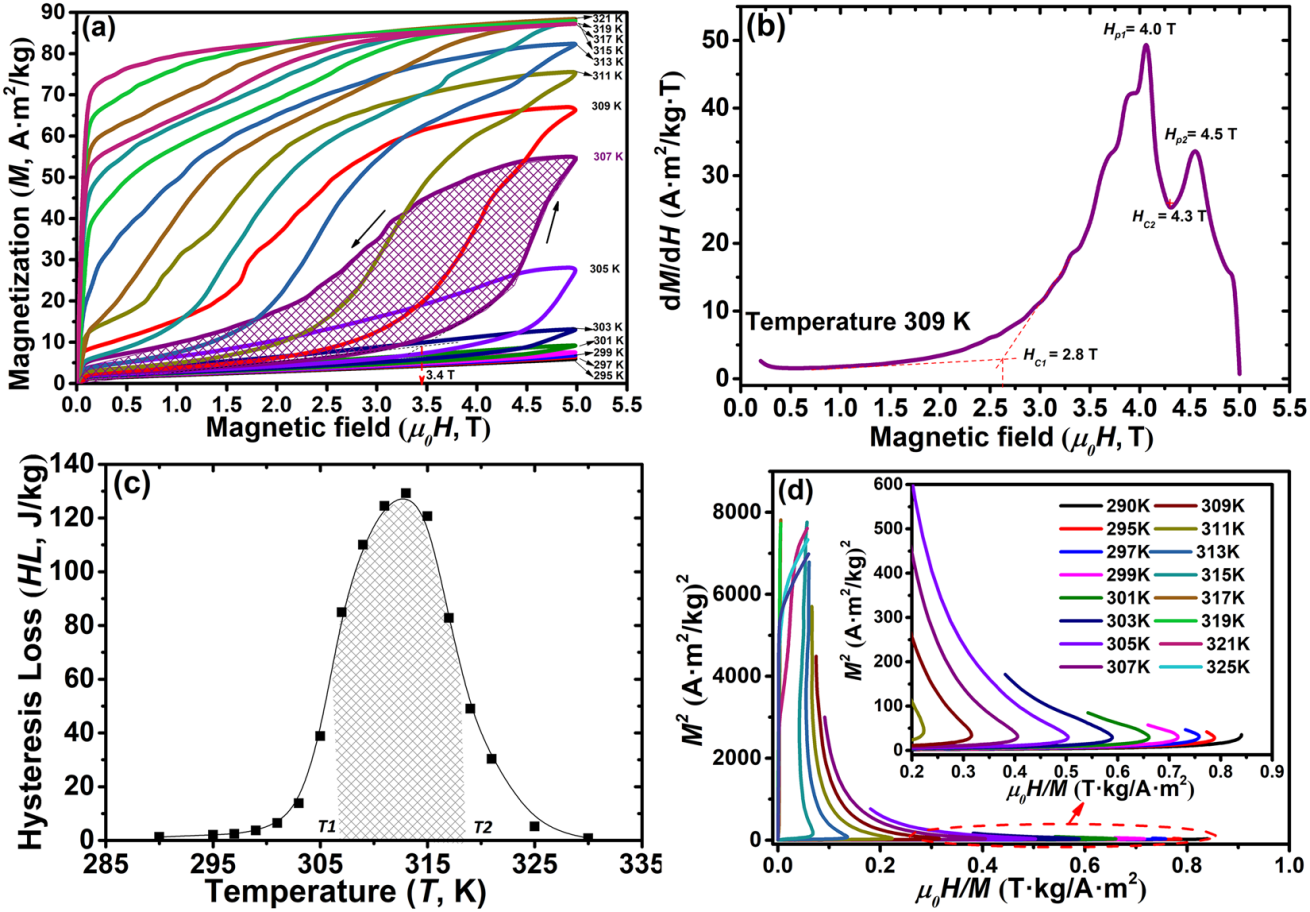
Magnetic transition behavior of the Co6 alloy. (**a**) Representative isothermal magnetization *M-H* loops under magnetic fields up to 5.0 T. (**b**) d*M*/d*H* curve measured at a temperature of 309 K showing two inflection points that indicate the occurrence of two successive magnetostructural transformations. (**c**) The temperature dependent hysteresis loss (*HL*) plot during the isothermal magnetization *M-H* cycling. The fitting line just acts as a guide for the eye. (**d**) Arrott (*M*^2^ *vs H/M*) plots revealing the appearance of the magnetic-field-induced austenite (MIA) at temperatures above 295 K.

The isothermal magnetization *M-H* loops for Co6 alloy at temperatures 290–330 K are shown in Fig. 6a. The plots were recorded under magnetic field up to 5.0 T and at a temperature interval *ΔT* = 2 K covering the *A*_*s*_ and *A*_*f*_. At a temperature of 295 K, far below *As*^*VSM*^ (314 K in Table 1), no austenite was induced by magnetic field up to 5.0 T. When test temperatures approach *As*, the magnetic-field-induced austenite (MIA) transformation gradually occurs, with MIA critical magnetic field decreasing with increasing temperatures. At 307 K, a large jump in magnetization at about 3.4 T (Fig. 6a), due to the occurrence of MIA, was observed. After removing the magnetic field, the sample transformed back to martensite, but with a small amount of field-induced austenite remaining because the test temperature 307 K was slightly higher than *M*_*f*_^*DSC*^ (304 K in Table 1)^59^. The d*M*/d*H* curve measured at 309 K shows two successive inflection peaks centered at 4.0 and 4.5 T (as shown in Fig. 6b), implying the appearance of the two successive magnetostructural transformations. The first inflection peak at *H*_*p1*_ = 4.0 T corresponds to the field-induced inverse martensite transformation 6 M + 10 M → A, and the second peak at *H*_*p*2_ = 4.5 T to the 10 M → A. The fact that *dM/dH* under 4.0 T is larger than that under 4.5 T implies that the phase transition resistance of the former is smaller than the latter one.

During isothermal magnetization *M-H* cycling, the hysteresis loss (*HL*) was determined by integrating the areas between magnetization and demagnetization branches (purple shaded area in Fig. 6a). The temperature dependence of *HL* is plotted in Fig. 6c. The average hysteresis loss (*AHL*), calculated by averaging the integral area under the temperature range of the full width at half maximum of the hysteresis peak$AHL={\int }_{T1}^{T2}\frac{f(HL)dT}{T2-T1},$ ($\mathrm{where}\,T1=306\,K,\,T2=318\,K$, as shown in Fig. 6c), was determined to be 99 J/kg under 5.0 T. This is comparable with those of the Ni-Mn-Sn-based alloys, i.e. *AHL* = 138 J/kg under 5.0 T in Ni_43_Mn_46_Sn_11_^17^ and *AHL* = 78 J/kg under 3.0 T in Ni_45_Co_5_Mn_40_In_2_Sn_8_^60^ alloys. In order to reveal the first-order martensite transformation (FOMT) temperature, Arrott plots (*M*^*2*^ *vs μ*_0_*H/M*) were derived as shown in Fig. 6d. The negative slopes can be detected at temperatures ≥295 K, suggesting the occurrence of the magnetic-field-induced austenite (MIA) formation^61^. Furthermore, the large degree of negative slope indicates that MIA is fast^18^, which is consistent with the *M-H* curves displayed in Fig. 6a.

### Magnetocaloric effects (MCE) of the Ni_40.6_Mn_43.3_Sn_10.0_Co_6.1_ (Co6) alloy

The MCE can be evaluated via *ΔS*_*M*_ calculated using thermodynamic Maxwell equation:

$$\Delta {S}_{M}(T,H)=S(T,H)-S(T,0)={\int }_{0}^{{H}_{\max }}(\partial M/\partial T)\,dH.$$

Based on isothermal magnetization data with a field interval of *ΔH*_*i*_ = 0.05 T at various temperatures (shown in Fig. 6a), the above partial derivative and integration may be numerically approximated to the equation:

$$\Delta {S}_{M}(T,H)={\sum }_{i}\frac{M({T}_{i+1},H)-M({T}_{i},H)}{{T}_{i+1}-{T}_{i}}\Delta {H}_{i}.$$

**Figure 7:**
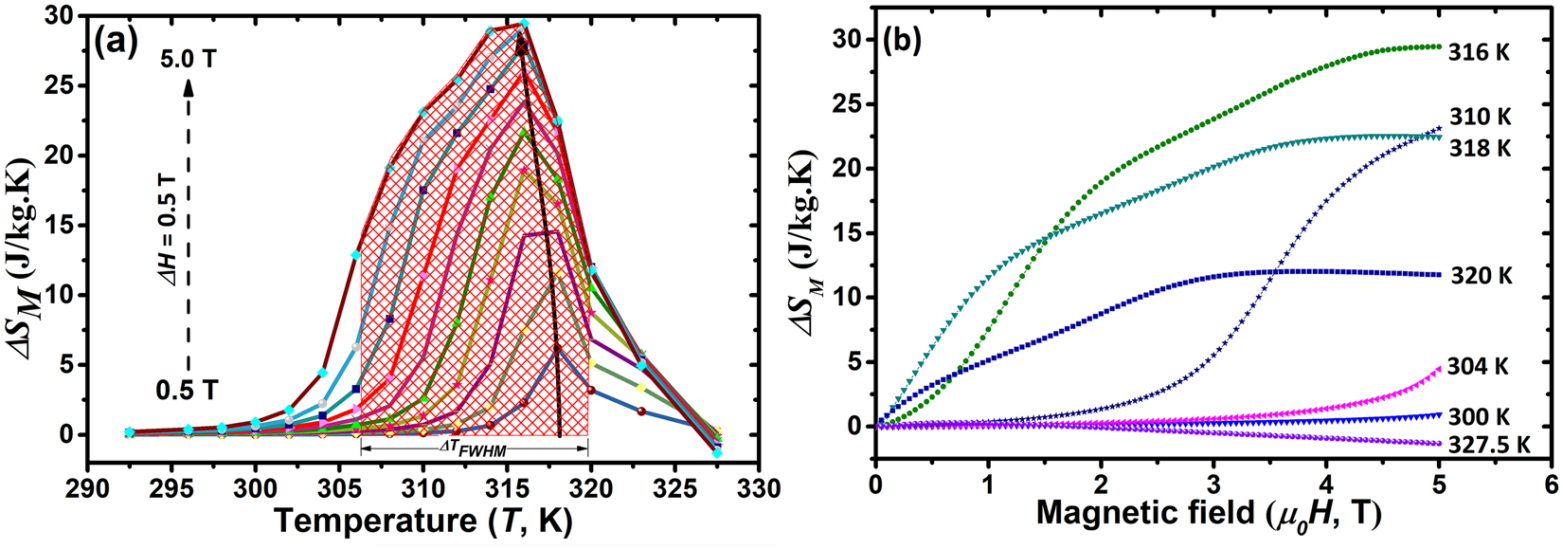
Magnetocaloric effects (MCE) of the Co6 alloy. (**a**) Selected temperature dependent magnetic entropy change (*ΔS*_*M*_) curves under magnetic fields *ΔH* = 0.5–5.0 T, (**b**) Magnetic entropy change *ΔS*_*M*_ as a function of the applied magnetic field at different temperatures in the reverse MT range.

The *ΔS*_*M*_ related to the FOMT for the Co6 alloy under *ΔH* = 0.5–5.0 T is plotted as a function of temperature displayed in Fig. 7a. The positive *ΔS*_*M*_ is attributed to inverse MCE originating from a magnetic-field-induced metamagnetic transition from the weak-magnetic martensite to the ferromagnetic austenite phase^2^. Additionally, the *ΔS*_*M*_ peak temperature exhibits a field-dependent behavior, i.e. shifting to lower temperatures with increasing magnetic fields.

As depicted in Fig. 6a, the magnetization saturates at lower fields with increasing temperature above *A*_*s*_. According to the Maxwell equation, further increase of the magnetic field contributes little contribution to *ΔS*_*M*_ when the saturation magnetization plots intersect, such as the temperatures of 317, 319 and 321 K in Fig. 6a. Therefore, the maximum *ΔS*_*M*_ $(\Delta {S}_{M}^{max})$ for a field change 5.0 T can be achieved at a specific temperature, i.e. 314 K here in Co6 alloy, where the adjacent *M-H* curves at 313 and 315 K do not intersect. For the present Co6 alloy, the $\Delta {S}_{M}^{max}$ reaches 29.5 J/kg·K under a magnetic field 5.0 T, with a working temperature span *ΔT*_*FWHM*_ (defined as the full width at half maximum of $\Delta {S}_{M}^{max}$) 306–320 K. The *ΔS*_*M*_ calculated from the Maxwell relation is compared with those from the Clausius-Clapeyron equation and DSC curves, as shown in Supplementary Materials TABLE S1. The $\Delta {S}_{M}^{max}$ is comparable to the value determined by Clausius-Clapeyron (32.9 J/kg·K), and both of them are also similar to the DSC results (*ΔS*_*tr*_^*endo*^ = 33.7 ± 0.2 J/kg·K and *ΔS*_*tr*_^*exo*^ = 32.2 ± 0.2 J/kg·K), implying that *ΔS*_*M*_ obtained by the Maxwell equation is acceptable in the present work without the occurrence of the so called “colossal” values around the transition temperature. In addition, the obtained $\Delta {S}_{M}^{max}$ in Co6 alloy is slightly higher than those of some other Co-doped Ni-Mn-based compounds whose $\Delta {S}_{M}^{max}$ were estimated using the loop-process method as well, such as Ni_50_Co_2_Mn_33_In_15_ (21.5 J/kg·K)^62^, Ni_45_Co_5_Mn_37.5_In_12.5_ (20 J/kg·K)^63^, Ni_44_Nb_1_Co_5_Mn_40_Sn_10_ (23 J/kg·K)^64^ and Ni_40_Co_10_Mn_40_Sn_10_ (27 J/kg·K)^13^. Furthermore, the present $\Delta {S}_{M}^{max}$ is also comparable to some rare-earth containing magnetic refrigerants such as Gd_5_Ge_2.3_Si_1.7_ (35.1 J/kg·K)^65^, MnAs_0.97_P_0.03_(33.4 J/kg·K)^66^ and MnFeP_0.8_Ge_0.2_ (34.3 J/kg·K)^67^.

On the other hand, MCE related to FOMT usually occurs in a narrow temperature range, for instance 1–5 K. By contrast, a broadened *ΔT*_*FWHM*_ ~14 K was obtained in the present Co6 alloy, which may be attributed to two aspects: 1) Two successive magnetostructural transformations (A → 10 M and A → 10 M + 6 M), 2) Magnetic-field-induced metamagnetic transition, i.e. the magnetic field dependence of the $\Delta {S}_{M}^{max}$ position. Here, *A*_*f*_^*5.0T*^ > *A*_*s*_^*0.02T*^ implies an incomplete reverse MT induced by a magnetic field of 5.0 T. In other words, the field-induced shift of the MT temperature cannot overcome MT width so that $\Delta {S}_{M}^{max}$ fails to saturate to *ΔS*_*tr*_. Whether a maximum of $\Delta {S}_{M}^{max}$ is reached under a field of 5.0 T needs further investigation in the near future. In Ni_45_Mn_36.7_In_13.3_Co_5_ alloy, a maximum $\Delta {S}_{M}^{max}$ = 7 J/kg K was achieved at a field of 4.0 T using “rectilinear modelization” by Recarte *et al*.^68^. While $\Delta {S}_{M}^{max}$ of the Ni_45.5_Co_4.5_Mn_37_In_13_ single crystal does not saturate up to 7.0 T using “curved modelization” adopted by Bourgault *et al*.^69^. Both approaches are equally consistent with the experimental data at a low magnetic field range (<4.0 T), whereas the “curved modelization” is better in reflecting $\Delta {S}_{M}^{max}$ at high fields. The MCE tends to reach a saturation value for fields above 5.0 T in Ni_51_Mn_33.4_In_15.6_ alloy by quasi-directly from isofield calorimetric measurements according to Stern *et al*.^70^ Fig. 7b shows *ΔS*_*M*_ - *T* plots at different temperatures in the reverse MT range. At low temperatures (i.e. *T* < 316 K), the field-induced fraction of austenite phase increases with the rise of temperature, which is responsible to a similar increase in *ΔS*_*M*_. At higher temperatures, where both martensite and austenite phases coexist at zero field, the field-induced austenite fraction and *ΔS*_*M*_ minify as the temperature increases. Furthermore, the negative slope of *ΔS*_*M*_ which can be clearly seen in Fig. 7b for high magnetic fields at 320 and 327.5 K, which is due to the contribution of this conventional direct MCE in the vicinity of *T*_*C*_^*A*^.

**Figure 8:**
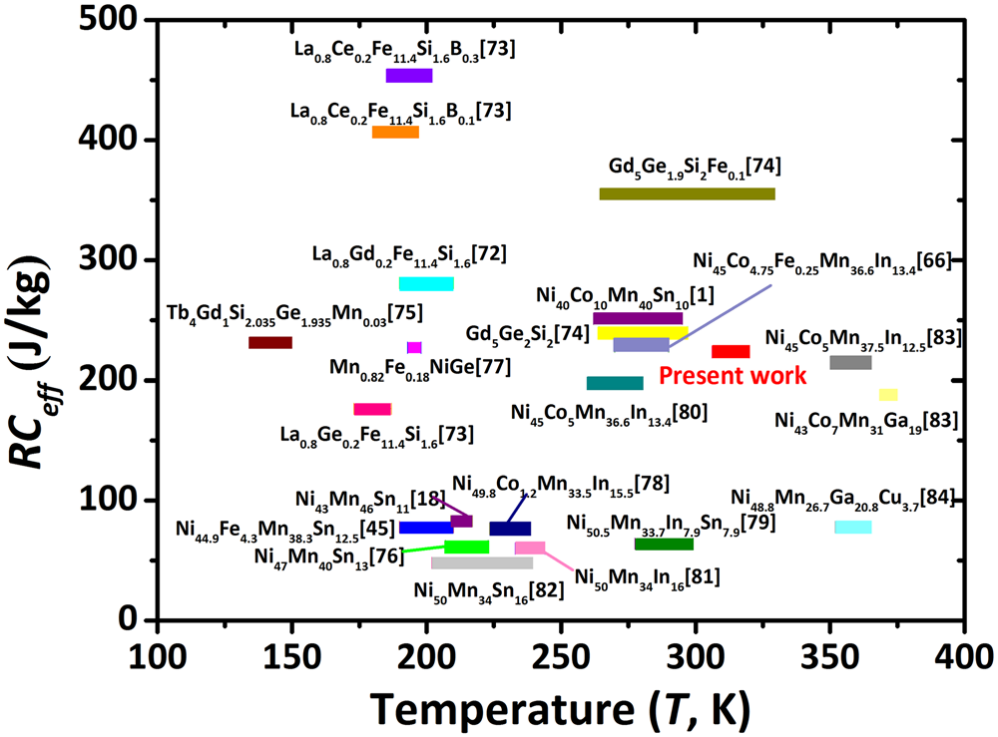
Effective magnetic refrigeration capacity *RC*_*eff*_ and working temperature span under a magnetic field of 5.0 T for the present Co6 alloy and some most studied magnetocaloric materials, such as La(FeSi)_13_-based^72,73^, Gd_5_(SiGe)_4_-based^74,75^, NiMn-based^1,18,45,63,76–84^ compounds. These alloys were selected because their magnetocaloric effects (MCE) are all related to the first-order martensite transformation and are tested around the magnetostructural transition temperatures.

The refrigeration capacity (*RC*), which represents the amount of heat transferring between the cold and hot reservoirs in a thermodynamic cycle, is defined as $RC={\int }_{{T}_{cold}}^{{T}_{hot}}|\Delta {S}_{m}(T)|dT$, where *T*_*cold*_ and *T*_*hot*_ are the corresponding temperatures at *ΔT*_*FWHM*_ of $\Delta {S}_{M}^{max}$. By numerically integrating the area under the *ΔS*_*M*_ -*T* curves between *T*_*cold*_ and *T*_*hot*_ (shaded area in Fig. 7a), the *RC* = 322 J/kg under a magnetic field change of 5.0 T was obtained in Co6 alloy. However, for evaluating the effective magnetic refrigeration capacity, the *AHL* should be deducted^18,45,71^. Therefore, a more reasonable criterion for assessing the cooling efficiency, the effective refrigeration capacity (*RC*_*eff*_) defined as *RC*_*eff*_ = *RC- AHL*, was adopted here. For comparison, the *RC*_*eff*_ values under a field of 5.0 T for the present work and some most studied magnetocaloric materials^1,18,45,63,72–84^ are summarized in Fig. 8. Noteworthily, the *RC*_*eff*_ of the present Co6 alloy reaches 223 J/kg, which is significantly larger than those of the stoichiometric Ni-Mn-based alloys^18,45,76,77,79,81–84^ and is comparable to those of some Co-doped Mn-rich Ni-Mn-based compounds^1,63,78–80,83^. Besides, the achieved *RC*_*eff*_ is also comparable to those rare-earth containing La(FeSi)_13_-based^72,73^ and Gd_5_(SiGe)_4_-based^74,75^ compounds. Of note is that the working temperature of the present alloy is slightly above 300 K, which is very encouraging for room-temperature magnetic refrigeration applications.

## Conclusion

The effect of Co doping on the martensite transformation, magnetic properties and magnetocaloric effects were investigated in Ni_47−x_Mn_43_Sn_10_Co_x_ (x = 0, 6, 11) alloys. The main conclusions may be drawn as follows:

1. The martensite transformation temperatures decreased and Curie temperature of austenite phase increased significantly with increasing Co concentration, broadening the temperature window for a higher magnetization of austenite: 13.5, 91.7 and 109.1 A·m^2^/kg for x = 0, 6 and 11, respectively.
2. Two successive magnetostructural transformations, i.e. A → 10 M and A → 10 M + 6 M, existed in the Ni_40.6_Mn_43.3_Sn_10.0_Co_6.1_ alloy whose magnetic-field-induced shift of austenite finishing temperature *ΔA*_*f*_*/ΔH* reached −2.6 K/T.
3. A sizable maximum magnetic entropy change $\Delta {S}_{M}^{max}$ 29.5 J/kg·K, a wide working temperature span *ΔT*_*FWHM*_ 14 K and a high effective refrigeration capacity *RC*_*eff*_ 223 J/kg were obtained in the Ni_41_Mn_43_Sn_10_Co_6_ alloy under a magnetic field of 5.0 T.

## Methods

### Sample and heat treatment

Ni_47−x_Mn_43_Sn_10_Co_x_ (x = 0, 6, 11, atomic percent) ingots, denoted as Co0, Co6 and Co11, respectively, were prepared by induction melting pure Ni (99.99%), Co (99.99%), Mn (99.9%) and Sn (99.99%) in an argon atmosphere and casting in a copper mold. The ingots were sealed in a quartz tube accompanied with Mn powder (to create a Mn vapor atmosphere in the tube) and Ti sheet (acted as oxygen getter), evacuated under vacuum at 10^−2^ Pa, annealed at 1173 K for 24 h and then quenched in iced-water. The composition of the specimens were determined by a Zeiss-SUPRA SEM equipped with an Oxford EDS, using 20 kV voltage, 97 µA emission current, 10 mm working distance, 50 µA probe current and >60 s data acquisition time duration. The composition measurement precision of the EDS was calibrated with chemical analysis results (ICP-OES) to be less than 0.5%.

### Composition and martensite transformation tests

The actual and nominal compositions of the alloys were shown in Table 1. The microstructure of the specimens was examined by a Zeiss-SUPRA SEM and an Olympus PMG3 optical microscope (OM) with a polarizing filter. The martensite transformation temperatures and Curie points were measured by differential scanning calorimetry (DSC) at heating and cooling rates of 10 K/min. XRD analyses were performed using Cu Kα radiation with a low-temperature chamber.

### Magnetic property and magnetocaloric effects (MCE) evaluation

Magnetization measurements were performed using a vibrating sample magnetometer (VSM) in a commercial Magnetic Property Measurement System (MPMS) of Quantum Design. Magnetization *vs* temperature (*M-T)* curves were recorded under magnetic fields 0.02 and 5.0 T with heating/cooling rates of 5 K/min and temperature range 200–400 K. Isothermal magnetization (*M-H)* curves were measured at different test temperatures (*T*_*test*_) from temperatures 290–330 K under an external magnetic field up to 5.0 T. In order to rule out the temperature and field history effects and thus avoid the spurious spike, the so-called loop process^82^ was performed before each *M-H* test. The detailed loop process was as follows: 1) The sample was initially zero-field-cooled down to 200 K to ensure a full weak-magnetic martensite state prior to recording each *M-H* cycle at a constant temperature, 2) Zero-field-heated to (*T*_*test*_−10) K at 10 K/min, then heated to *T*_*test*_ at 1 K/min and finally maintained at the *T*_*test*_ temperature for 2 min before starting the *M-H* cycle.

### Data availability statement

The datasets generated during and/or analyzed during the current study are available from the corresponding author on reasonable request.

## Electronic supplementary material

Supplementary Materials
