# Supplementary material for: Enhanced magnetocaloric effect in Ni-Mn-Sn-Co alloys with two successive magnetostructural transformations
Source: Sci Rep. 2018 May 29;8:8235. doi: 10.1038/s41598-018-26564-5 (PMC5974186; doi:10.1038/s41598-018-26564-5)
Supplement: Supplementary file 1 — Supplementary Materials [file 41598_2018_26564_MOESM1_ESM.pdf]

Supplementary Materials

Enhanced magnetocaloric effect in Ni-Mn-Sn-Co alloys with two successive magnetostructural transformations

Xuexi Zhang*, Hehe Zhang, Mingfang Qian, Lin Geng

School of Materials Science and Engineering, Harbin Institute of Technology, Harbin 150001, China

*Corresponding author. Tel: 86-451-86415894, Fax: 86-451-86413921, E-mail: xxzhang@hit.edu.cn

Microstructure, entropy change and crystal structure of the Co6 alloy

Back scattered electron (BSE) image and X-Ray diffraction (XRD) pattern of the annealed $Ni_{40.6}Mn_{43.3}Sn_{10.0}Co_{6.1}$ (Co6) alloy in austenite state were carried out, as shown in Fig. S1. The BSE micrograph of the annealed Co6 alloy is shown in Fig. S1a. It can be seen that the alloy is homogeneous except some micro-pores created during solidification, i.e. no Co-rich secondary phase can be observed. The high temperature XRD pattern shown in Fig. S1b confirms that only austenite phase is detected in the Co6 alloy, which is consistent to the BSE result.

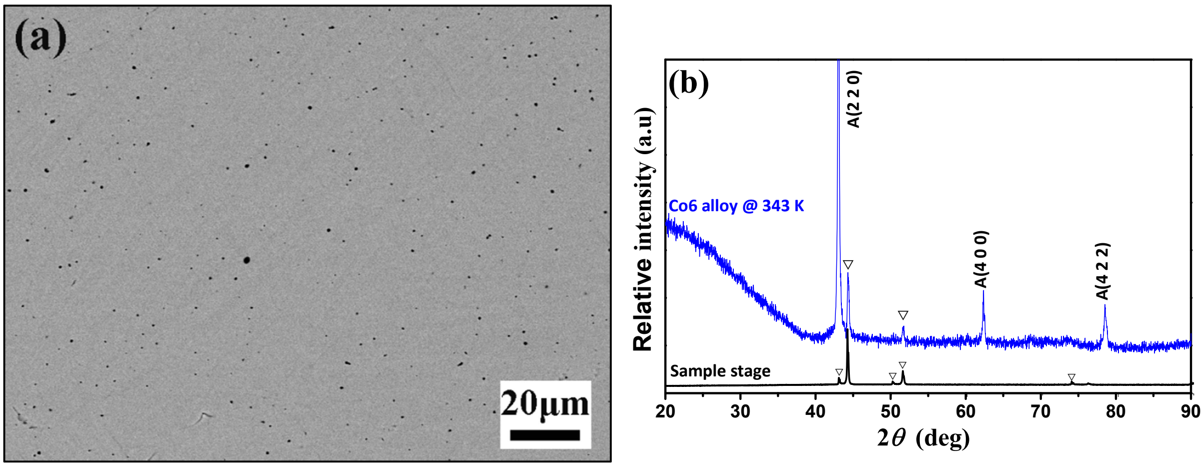

Fig. S1 (a) Back scattered electron (BSE) micrographs of Co6 alloy at room temperature. (b) X-ray diffraction (XRD) patterns at 343 K (> A_f) for Co6 alloy. Signal of the sample stage was also observed since the sample stage cannot cover the whole surface of the sample stage.

For the present Co6 alloy, the first-order martensite transformation may be induced by thermal and magnetic fields. The latent heat released or absorbed during this structural

transformation reflects its caloric effect. Here the magnetic entropy change (ΔSM) was calculated using Maxwell equation (MQ), Clausius-Clapeyron equation (C-C) and differential scanning calorimetry (DSC) methods. The results were summarized in TABLE SI.

The Maxwell equation can be described as:

$$\Delta S_M(T,H) = S(T,H) - S(T,0) = \int_0^{H_{\max}} (\partial M / \partial T) dH \tag{1}$$

Based on isothermal magnetization (*M*-*H*) data with a field interval of ΔH_i = 0.05 T at various temperatures, the above partial derivative and integration of equation (1) may be numerically approximated to equation (2):

$$\Delta S_M(T,H) = \sum_i \frac{M(T_{i+1},H) - M(T_i,H)}{T_{i+1} - T_i} \Delta H_i \quad . \tag{2}$$

The Clausius-Clapeyron equation used for the determination of the entropy change is:

$$-\mathrm{d}T_M/dH = \Delta M/\Delta S_{tr} \tag{3}$$

From equation (3), ΔS_{tr} can be obtained using ΔM under 5.0 T as the saturation magnetization difference and $\Delta A_S/\Delta H$ (-2.6 K/T) as $\mathrm{d}T_M/dH$.

The differential scanning calorimetry (DSC) relation for calculating ΔS_{tr}^{endo} is:

$$\Delta S_{tr}^{endo} = \Delta H_{endo}/T_t \tag{4}$$

where $T_t = (A_s^{DSC} + A_f^{DSC})/2$.

$$\Delta S_{tr}^{exo} = \Delta H_{exo}/T_t \tag{5}$$

where $T_t = (M_s^{DSC} + M_f^{DSC})/2)$.

The enthalpy changes ΔH_{endo} and ΔH_{exo} are obtained by averaging the values of repeated integrating the heat flow curves.

TABLE SI Maximum magnetic entropy change (ΔS_M) values determined by Maxwell equation (MQ), Clausius-Clapeyron equation (C-C) and differential scanning calorimetry (DSC) methods.

Methods	MQ	C-C	DSC^{endo}	DSC^{exo}
			J/kg K	
Entropy change (ΔS_M)	29.6*	32.9	33.7±0.2	32.2±0.2

**Note:* The ΔS_M corresponds to a magnetic field of 5.0 T.

A fan-shaped Co6 sample with a diameter of 10 mm and inner angle about 150° was

used to study the martensite transformation sequence on the Bruker D8 ADVANCE in-situ X-ray diffraction (XRD) with a low-temperature chamber. The sample platform has a size of 12×16 mm^2. To reduce the intensity of the signal caused by the sample platform, amorphous ceramic powders were filled around the specimen in the groove of the sample platform.

For the martensite transformation study during cooling process, the sample was heated to a temperature of 343 K and kept at this temperature for 10 min, and then it was cooled to 313 K with a cooling rate of 1 K/min. After holding at this temperature for 5 min to reach a stable temperature state, the X-Ray scan was started with a scan rate of 2°/min. During the scanning process, the temperature fluctuation was found to be less than 1 K. After the scan, the sample was cooled at a rate of 1 K/min to a second test temperature, hold for 5 min and subjected to a second X-Ray scan. This process was repeated at temperatures of 343-173 K during cooling process. The data were recorded at a temperature interval 10 K (except for 223 K and 173 K). In the diffraction patterns of the alloy sample, there are two peaks from the sample holder center at 2θ = 44.2° and 51.5°, as shown in Fig. S1b. Fig. S2 displays a diffraction pattern of the Co6 alloy at a temperature of 173 K. The theoretically calculated diffraction peaks of the 6M and 10M martensite phases[1] were also shown in Fig. S2.

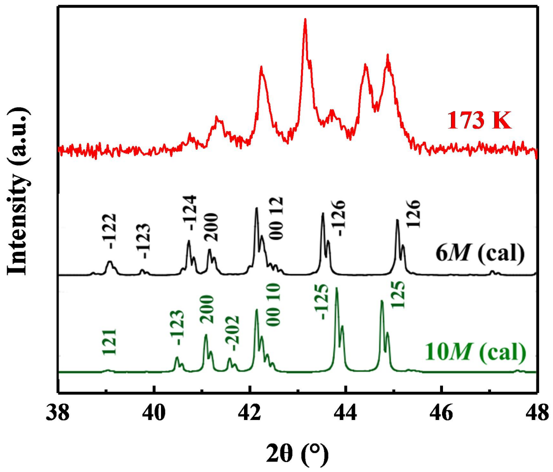

Fig. S2. X-Ray diffraction (XRD) pattern of the Co alloy sample at 173 K and the calculated patterns of 6M and 10M from Ref. [1].

Reference

1 Umetsu, R. Y., et al. Kinetic arrest behavior in martensitic transformation of NiCoMnSn metamagnetic shape memory alloy. J. Alloy. Compd. **509,** 1389-1393 (2011).
